# Trauma support in your pocket

**DOI:** 10.1093/eurpub/ckac131.175

**Published:** 2022-10-25

**Authors:** M Thell, K Edvardsson, G Warner

**Affiliations:** Department of Public Health and Caring Sciences, Uppsala University, Uppsala, Sweden

## Abstract

**Background:**

Research indicates that technological approaches to the delivery of trauma-based support can help address structural and perceptual barriers. Yet, there is a lack of research on digital trauma support for children and adolescents. A systematic review of trauma apps identified 2 of 69 were for children, of which neither were evidence based. The aim of this project was to develop a trauma support app, co-designed with and for children and young people, based on a community-based group support program called Teaching Recovery Techniques.

**Methods:**

In a series of workshops utilising the Design Studio method, a team of 7 young people between 14-20 years (3 boys and 4 girls) co-created the app. The team were recruited via youth organisations and self-reported having experienced (undisclosed) trauma. The techniques presented in the manual have been prioritised via stepwise consideration of content session by session. Prototypes were developed based on the generated ideas and shared with the team for feedback.

**Results:**

Contributions of the young people to the design can be considered in three categories: mechanics, dynamics and aesthetics. Mechanics are the rules and interactions that inform the structure of the app. Dynamics refers to what the user can actually see, e.g. the outcome when the user presses a button. Aesthetics relate to the desirable emotional responses evoked in the user when they interact with the app. Beyond influence on basic aesthetics, such as fonts and symbols, the young people actively contributed to the user experience and gave great consideration to the emotional responses that could be evoked.

**Conclusions:**

Young people with personal experience of trauma can actively engage in the development of a digital trauma intervention. Design Studio was an effective method for the co-creation process, enabling the generating and converging of young peoples’ ideas.

**Key messages:**

• Co-creating an app for trauma support with youth with experience of trauma was a feasible process for both researchers and youth.

• Using Design Studio, a collaborative workshop method, facilitated the co-creative process, allowing the youth to contribute to the mechanics, dynamics and aesthetics factors of the app.

